# Impact of Gestational Diabetes Mellitus on Fetal Growth and Nutritional Status in Newborns

**DOI:** 10.3390/nu16234093

**Published:** 2024-11-27

**Authors:** Karolina Karcz, Barbara Królak-Olejnik

**Affiliations:** Department of Neonatology, Wroclaw Medical University, 50-367 Wrocław, Poland; barbara.krolak-olejnik@umw.edu.pl

**Keywords:** gestational diabetes mellitus, fetal growth, neonatal nutritional status, perinatal outcomes, adipokines

## Abstract

Background: Gestational diabetes mellitus (GDM) is one of the most prevalent complications associated with pregnancy, exhibiting a gradual rise in prevalence worldwide. Given the potential for numerous short- and long-term complications for both mother and child, patients diagnosed with GDM require individualised treatment to compensate for metabolic abnormalities and ultimately reduce the risk of the known adverse consequences of impaired glucose tolerance. Methods and results: The manuscript presents a summary of the current knowledge on changes in maternal metabolism during physiological pregnancy and pregnancy complicated by gestational diabetes. Furthermore, the article provides a synthesis of the findings from recent research examining the impacts of gestational diabetes and the therapeutic modalities employed on the nutritional status of the fetus and neonate. Additionally, the review elucidates the function of the placenta and placental hormones in fetal development, as well as the impact of hyperglycemia, insulin resistance and adipokines on fetal and neonatal nutritional programming and predisposition to metabolic complications in adulthood. Conclusions: The metabolic environment, resulting from abnormal glucose tolerance during pregnancy, exerts a particularly significant impact on fetal growth and, consequently, on the birth weight and fat mass of the newborn infants. This is a pivotal factor influencing the nutritional and metabolic programming of the developing fetus, predisposing the individual to the development of metabolic complications throughout their lifetime.

## 1. Introduction

Gestational diabetes is one of the most prevalent metabolic complications of pregnancy. It is currently estimated that gestational diabetes accounts for approximately 90% of all cases of diabetes in pregnancy, affecting approximately 5.4% of women in Europe and 3.4% of women in Poland [1]. The rising prevalence of gestational diabetes can be attributed to a number of factors, including genetic predisposition and the increasing prevalence of obesity among women of childbearing age, as well as the later age at which they decide to have offspring.

The clinical significance of gestational diabetes necessitates a multifaceted approach to its early diagnosis and proper treatment, given the risk of numerous short- and long-term health complications for both the mother and her child. It is currently understood that infants born to mothers with diabetes are at an elevated risk for a range of potential complications, many of which are transient and may resolve without significant consequence. These include hypoglycemia, hyperbilirubinemia, hypocalcemia, hypomagnesemia, polyglobulia, respiratory disorders or respiratory failure during the postpartum adaptation period, and cardiomyopathy. Additionally, there has been an increase in the incidence of macrosomia, polyembryony, stillbirths, perinatal injuries, and surgical deliveries. In the long-term aspect, the occurrence of diabetes in the mother during pregnancy has been linked to an increased risk of developing obesity, abnormal glucose tolerance, and diabetes in her offspring. Furthermore, in the case of uncontrolled diabetes, there is an increased risk of neurodevelopmental disorders. However, the majority of the aforementioned complications can be attributed to overnutrition and fetal macrosomia, which are primarily caused by maternal disturbances in glucose and fat metabolism during pregnancy [2,3,4].

The concept of nutritional programming suggests that a child’s nutritional status during the first 1000 days after conception has a significant impact on their neurological development, lifelong mental health, and risk programming for the development of obesity and hypertension [5].

## 2. The Metabolic Alterations That Occur in the Body During Physiological Pregnancy

To meet the increased nutrient requirements of the mother during pregnancy, it is necessary to provide her with energy stores that will be used during various conditions during pregnancy, childbirth, and lactation. In addition, it is essential to ensure an adequate supply of nutrients for optimal fetal growth and development. These requirements necessitate adaptive changes in the metabolic processes of the maternal body [6,7].

### 2.1. Carbohydrate Metabolism During Physiological Pregnancy

The initial stage of pregnancy is typified by normal glucose tolerance, which at times demonstrates improvement. Peripheral insulin sensitivity and basal hepatic glucose production are maintained at comparable levels to those observed prior to pregnancy. This is significant because consistent metabolic conditions, influenced by cortisol, estrogen, and progesterone, facilitate lipogenesis and fat storage [8,9]. As gestation progresses, there is an increase in insulin secretion, both in response to nutrient supply and at the basal level. This is accompanied by a slight deterioration in glucose tolerance, indicating an increase in peripheral tissue insulin resistance. It is estimated that basal fasting glucose levels in the third trimester are approximately 10 mg/dL lower than in the second trimester, and insulin levels are approximately twice as high as in non-pregnant women. Hepatic gluconeogenesis increases in proportion to maternal weight, yet per unit of body weight, production remains constant. Postprandial glucose concentrations reach considerably elevated values, exhibiting a prolonged glucose peak. In contrast to peripheral tissues, liver cells remain sensitive to increasing insulin concentrations, which results in the suppression of gluconeogenesis. The alterations in carbohydrate metabolism are intended to satisfy the increasing requirements of the placenta and fetus.

In a pregnant woman, each meal elicits a complex cascade of hormonal responses. An elevation in blood glucose levels precipitates a secondary secretion of pancreatic insulin, glucagon, somatomedins, and adrenal catecholamines, thereby ensuring that the mother and fetus have access to a sufficient, yet not excessive, supply of glucose. Furthermore, there is a proclivity towards hypoglycemia between meals and during sleep. This is due to the increase in plasma volume that occurs in the early stages of pregnancy and, in more advanced pregnancy, the continuous transport of glucose to the fetus via the placenta. The prevalence of hypoglycemia rises in tandem with the duration of pregnancy and the mounting demands of the fetus. Furthermore, the interval between meals results in a more pronounced decline in glucose levels, with approximately 30% greater hepatic gluconeogenesis and ketogenesis, as well as a reduction in plasma free amino acid concentrations. Furthermore, the increased carbohydrate requirements of the fetus and placenta result in augmented endogenous glucose production, namely gluconeogenesis and glycogenolysis. Additionally, there is a gradual increase in the amount of insulin secreted during the second and third trimesters of pregnancy, which is attributed to insulin resistance caused by the activity of placental hormones, namely estrogen, progesterone, and chorionic somatotropin [6,7].

### 2.2. Lipid Metabolism During Physiological Pregnancy

Pregnancy with a physiological course is also distinguished by an elevation in maternal serum lipids, which affects all lipid fractions, with triglycerides exhibiting the most pronounced increase. Moreover, functional and morphological alterations in adipocytes are observed, including cell hypertrophy and an increase in the number of insulin receptors, which facilitate fat deposition during the first and second trimesters of pregnancy. This process is regulated by the increased concentrations of insulin, which stimulate lipogenesis and inhibit lipolysis, as well as by the activity of estrogen and progesterone. In the third trimester of pregnancy, the process of lipogenesis ceases. The accumulated fat stores serve as the primary source of energy for the mother, facilitating the oxidation of fatty acids and enabling the efficient transport of glucose and amino acids to the fetus [6,10].

In the advanced stages of pregnancy, the administration of chorionic somatomammotropin has been observed to facilitate the breakdown of adipose tissue, leading to an increase in the concentration of fatty acids and glycerol in the maternal plasma [9]. Furthermore, augmented maternal insulin resistance in conjunction with lipolysis of peripheral adipose tissue gives rise to elevated lipoprotein concentrations and an augmentation of lipoprotein triglycerides, including VLDL, HDL, and LDL. The transition from an anabolic to a catabolic state facilitates the utilization of lipids as a maternal energy source while preserving glucose and amino acids for the fetus. In the event of starvation, there is a rapid redirection of maternal metabolism towards fat oxidation, with the utilization of ketones as an energy substrate [11,12]. The supply of free fatty acids, as well as fatty acids and cholesterol carried in maternal lipoproteins, is crucial for fetal growth and development. Maternal lipoproteins do not traverse the placenta directly; however, their constituents may be conveyed to the fetus via interactions with particular lipoprotein receptors, lipases, and fatty acid-binding transport proteins [9,11].

### 2.3. Protein Metabolism During Physiological Pregnancy

The concentration of amino acids in the maternal blood serum exhibits fluctuations throughout the course of pregnancy. During the first trimester, it declines in tandem with the expansion of maternal blood volume. In the second trimester, however, it attains a relatively stable plateau, exhibiting a reduction of approximately 1 g/L compared to the pre-pregnancy level. By the 20th week of gestation, total albumin concentration also declines, resulting in a reduction in oncotic pressure and an increased propensity for peripheral edema. The reduction in maternal serum amino acid concentrations is attributable not only to diuresis, but also to their enhanced active transport by specialized transporters in accordance with the concentration gradient across the placenta. Pregnancy is a period of primarily facilitated anabolism, which results in limited protein breakdown and consumption. However, the shorter intervals between meals compared to the pre-pregnancy period result in the utilization of amino acids for fetal glucose production [6].

## 3. The Metabolic Changes That Occur in the Body During Pregnancy, When Gestational Diabetes Is Present

The current understanding of gestational diabetes is that it arises from a combination of factors, including excessive insulin resistance in peripheral tissues, insufficient insulin secretion, interactions between hormones produced by the placenta and those produced by adipose tissue and other peripheral tissues, pro-inflammatory cytokines, and genetic predisposition. Women diagnosed with gestational diabetes exhibited impaired insulin secretion in response to hyperglycemia, which may indicate pancreatic beta-cell dysfunction, as well as impaired suppression of hepatic glucose production in response to insulin action. Furthermore, impaired activation of the insulin signaling pathway was observed. Furthermore, these women exhibit elevated fasting serum insulin levels [6,13].

The development of insulin resistance in women with gestational diabetes also results in the formation of lipid abnormalities, which are characterized by elevated serum concentrations of fatty acids and triglycerides in comparison to pregnant women with normal glucose tolerance. The precise impact of insulin and insulin resistance on amino acid metabolism remains unclear [6,11].

With regard to fetal growth, it has been demonstrated that the incidence of macrosomia in pregnancies complicated by gestational diabetes mellitus is associated with maternal blood levels of free fatty acids and triglycerides, as well as postprandial glycemic values in the case of glucose. Postprandial hyperglycemia is regarded as the primary factor influencing accelerated fetal growth. Maternal hyperglycemia results in fetal hyperglycemia and hyperinsulinemia, which causes excessive fetal growth, particularly in regard to adipose tissue development and the ratio of head circumference to chest circumference. Additionally, elevated triglycerides and amino acids may contribute to fetal macrosomia by stimulating the secretion of insulin and other growth factors. It is noteworthy that pregnancies complicated by gestational diabetes have been demonstrated to exhibit augmented placental transport of glucose, fatty acids, and amino acids [6,11,13]. A comprehension of these alterations is of paramount importance for the effective management of GDM and the minimization of unfavorable pregnancy outcomes.

### 3.1. Hyperglycemia: Consequences for the Maternal, Fetal, and Newborn Populations

Hyperglycemia in pregnancy, irrespective of the underlying etiology of diabetes, is associated with an elevated risk of both long-term and short-term complications for both the mother and the neonate. Prolonged uncontrolled diabetes in the initial stages of pregnancy may result in embryo loss prior to implantation or at an early developmental stage. In light of the potential complications, maternal hyperglycemia is regarded as a teratogenic factor [12]. In contrast to the abnormal fetal growth observed in the early stages of pregnancy, the subsequent two trimesters are characterized by excessive glucose transport across the placenta, premature maturation and secretion of pancreatic insulin, and hyperinsulinemia in the developing fetus. These factors contribute to accelerated development and tissue hypertrophy, among other effects [9,12]. Clinical abnormalities in newborns of mothers with pre-pregnancy and gestational diabetes are common. These include abnormal physical development of the fetus, such as macrosomia (approximately 20%) and hypotrophy. Furthermore, there is an increased risk of perinatal hypoxia and respiratory disorders during the postnatal adaptation period, as well as a higher incidence of perinatal trauma, developmental defects, and metabolic, electrolyte, hematological, neurological disorders, and hyperbilirubinemia. These complications are more prevalent in mothers with vascular complications of diabetes, accounting for approximately 4% of cases [14,15]. In women, unregulated glucose metabolism is associated with an elevated risk of vascular complications, including hypertension, retinopathy, nephropathy, coronary artery disease, and neuropathy. Additionally, the percentage of pregnancies that are terminated by cesarean section is also increasing [14,15].

The molecular mechanisms of hyperglycemia-induced tissue damage include the overproduction of reactive oxygen species, the activation of protein kinase C, the alteration of arachidonic acid metabolism, leading to the abnormal synthesis of prostaglandins and nitric oxide, the increased production of advanced glycation end products, and the excessive production of polyols [12].

### 3.2. Lipid Metabolism in Women with Pregnancies Complicated by Diabetes Mellitus

Women with gestational diabetes mellitus (GDM) exhibit elevated triglyceride concentrations throughout all trimesters of pregnancy. Similarly, an increase in total cholesterol concentrations has been demonstrated, accompanied by equal or lower LDL concentrations and increased oxidation compared to women with physiologic pregnancies [11]. Primary abnormalities in carbohydrate metabolism in women with GDM give rise to other metabolic abnormalities, primarily involving lipid metabolism.

### 3.3. Maternal Dyslipidemia and Its Impact on the Course of Pregnancy

In comparison to a typical pregnancy, an elevation in maternal triglyceride levels during the initial stages has been linked to an increased likelihood of developing preeclampsia, gestational diabetes mellitus (GDM), preterm labor, and a higher relative fetal weight at the time of delivery. In observational studies, low maternal HDL cholesterol levels at the beginning of pregnancy have also been associated with an increased risk of later development of GDM. Additionally, higher HDL levels have been correlated with lower rates of preterm labor. There is scientific evidence indicating that elevated triglyceride levels and diminished HDL cholesterol are associated with unfavorable maternal and neonatal outcomes in women without diabetes mellitus [8,11].

Furthermore, a robust correlation has been established between prenatal exposure to maternal obesity and the likelihood of developing obesity in childhood. In comparison to women with a normal BMI, pregnant women with a high BMI have been observed to exhibit elevated fasting blood glucose and glucose load test values (though not meeting the criteria for a diagnosis of GDM or PDM), elevated triglyceride and VLDL levels, and reduced HDL-C cholesterol levels. Furthermore, the extent of gestational weight gain (GWG) was found to be a contributing factor to excessive fetal growth. Accordingly, there is an increasing body of evidence to suggest that not only maternal glucose concentration, but also triglyceride (TG) and free fatty acid (FFA) concentrations, influence fetal fat gain [9].

### 3.4. Insulin and Insulin Growth Factors (IGF) and Their Impact on Pregnancy Outcomes

In addition to its role in metabolic regulation, insulin in the fetus functions as a potent growth factor for somatic tissues. For this reason, insulin and associated growth factors, including IGF-1 and IGF-2, are implicated in the fetal overgrowth that is a characteristic of GDM. Elevated fetal insulin levels stimulate the processes of protein synthesis and lipogenesis, which in turn result in augmented tissue growth and the accumulation of adipose tissue. Furthermore, it has been demonstrated that the deposition of glycogen and fat in fetal tissues has been promoted by insulin. Furthermore, insulin plays a role in the proliferation and differentiation of muscle, adipose, and connective tissues, which contributes to disproportionate growth, particularly in the fetal shoulders, trunk, and abdomen [16,17,18,19,20].

Considering Insulin-like growth factors, IGF-1 is primarily regulated by nutrient availability and insulin, and it mediates many of insulin’s growth-promoting effects. It stimulates cell proliferation and differentiation in fetal tissues and supports overall somatic growth. While IGF-2 exhibits reduced responsiveness to insulin in comparison to IGF-1, it plays a pivotal role in placental and early fetal development, particularly in the promotion of trophoblast proliferation and placental nutrient transport [16,17]. It needs to be emphasized that IGF-1 and IGF-2 are locally produced in fetal tissues and act in an autocrine or paracrine manner to promote growth. In GDM, elevated insulin levels lead to increased production of these factors in tissues such as muscle, bone, and adipose, which in turn results in accelerated growth. What is more, the development of the placenta is supported by insulin and IGF-2, which may potentially alter its capacity to transfer nutrients and exacerbate fetal overgrowth [18,19].

Persistent alterations in the IGF-1 signaling pathway resulting from hyperinsulinemia may influence postnatal growth patterns and predispose offspring to metabolic syndromes, including obesity and type 2 diabetes. Furthermore, dysregulation of the insulin and IGF pathways may also have epigenetic consequences, modifying gene expression related to growth and metabolism [20].

## 4. The Role of Placenta and the Changes That Occur in Its Function

The placenta plays a pivotal role in orchestrating metabolic and endocrine adaptations during pregnancy. This is achieved through the production of hormones and signaling molecules, including growth hormones, lactogenic hormones, leptin, and adipokines [21,22,23]. In GDM, these processes are dysregulated, resulting in heightened insulin resistance, altered nutrient transport, and inflammation. It is of the utmost importance to gain an understanding of these changes in order to effectively manage GDM and reduce the likelihood of adverse maternal and fetal outcomes [22,23,24]. In normal pregnancy, Placental Growth Hormone (PGH) regulates maternal glucose metabolism by inducing insulin resistance, ensuring an adequate supply of glucose to the fetus. PGH replaces maternal pituitary growth hormone by mid-pregnancy and reduces maternal glucose uptake by enhancing insulin resistance in peripheral tissues. However, in GDM pregnancies, PGH levels are often elevated, exacerbating insulin resistance and hyperglycemia [21,22].

Placental Lactogen (hPL) functions to promote lipolysis, facilitating the mobilization of free fatty acids as an energy source for the mother whilst conserving glucose for the fetus. Furthermore, it contributes to the proliferation and functional adaptation of maternal pancreatic β-cells, thereby increasing insulin secretion. Nevertheless, in cases of GDM, the adaptive response of the β-cells may be inadequate, resulting in insufficient insulin secretion despite elevated insulin resistance [21,22].

In a typical pregnancy, prolactin plays a role in the expansion of maternal β-cell mass and enhances insulin production, which in turn aids in maintaining glucose homeostasis. However, in pregnancies complicated by GDM, a reduction in β-cell plasticity and responsiveness to prolactin may impair glucose regulation [23,24].

From a physiological standpoint, placental leptin plays a role in regulating maternal appetite and energy expenditure, as well as influencing insulin sensitivity. However, the overproduction of leptin by the placenta in cases of GDM can result in leptin resistance, thereby further contributing to the dysregulation of metabolism [21,23,24].

Adipokines generally regulate the processes of inflammation and insulin sensitivity. Adiponectin enhances insulin sensitivity, whereas resistin and pro-inflammatory cytokines (e.g., TNF-α) are associated with insulin resistance. In gestational diabetes mellitus (GDM), there is frequently an imbalance in the levels of adipokines, with a reduction in adiponectin and an increase in pro-inflammatory adipokines, which amplify insulin resistance [23,24].

It is evident that placental functional changes in gestational diabetes, such as vascularization and trophoblast dysfunction, have the potential to impair the delivery of nutrients and oxygen to the fetus [25]. This can subsequently affect fetal growth patterns, including macrosomia or intrauterine growth restriction. Additionally, altered transporter expression may result in excessive nutrient transfer, such as glucose and lipids, to the fetus [21,22]. Enhanced glucose transport via GLUT1 in the placenta can result in fetal hyperglycemia and hyperinsulinemia, which in turn predisposes the fetus to excessive growth (macrosomia) and increased fat deposition. An alteration in lipid metabolism may result in an increased transfer of free fatty acids to the fetus, which could in turn lead to an increase in adiposity and an elevated risk of developing long-term metabolic complications [22,25]. The excessive production of PGH, hPL and leptin serves to exacerbate maternal insulin resistance. The dysregulation of cytokines and adipokines has been demonstrated to increase systemic inflammation and impair metabolic adaptation [21,22,25].

## 5. Non-Pharmacological and Pharmacological Control of the Severity of Metabolic Disorders in the Course of Gestational Diabetes

The objective of gestational diabetes treatment is to reduce the risk of complications during pregnancy, such as pregnancy-induced hypertension, excessive fetal growth, maternal hyperglycemia or hypoglycemia. Additionally, the aim is to reduce the risk of complications in the perinatal period, including operative delivery, preterm delivery, perinatal trauma to the newborn, and metabolic disorders during postnatal adaptation in the newborn. Furthermore, the objective is to reduce the risk of distant health complications in the mother and child. The primary approach to managing gestational diabetes is a combination of dietary modifications, structured physical activity, regular monitoring of glycemic levels, and, if necessary, insulin therapy. The utilization of oral antidiabetic medications during the gestational period is not currently endorsed as a standard of care [1,26].

### 5.1. Non-Pharmacological Control of Gestational Diabetes

Dietary management constitutes a fundamental aspect of the treatment of GDM. The principal objective is to regulate blood glucose levels and avert maternal hyperglycemia. It is of the utmost importance to maintain a controlled intake of carbohydrates in order to prevent fluctuations in blood sugar levels. It is recommended that women with GDM focus on low glycemic index (GI) foods, which have been shown to cause slower rises in blood glucose level. The consumption of smaller, more frequent meals has been demonstrated to assist in stabilizing blood sugar levels, thereby preventing the occurrence of significant fluctuations. A balanced diet should comprise complex carbohydrates, lean proteins and healthy fats (such as those from avocados, olive oil, and nuts), which can help to improve satiety and to maintain a steady release of glucose into the bloodstream [26,27,28].

In fact, both elevated maternal mean fasting and postprandial blood glucose levels, as well as exceedingly low glycemic levels, have been associated with complications. An increased risk of complications during labor, as well as an elevated risk of obesity during the child’s lifespan, and elevated blood sugars have been identified as being associated with one another. Similarly, excessive hypoglycemia has been linked to maternal hypoglycemia, which is associated with a reduction in fetal birth weight. Inadequate caloric intake has the potential to result in elevated levels of third trimester ketonemia, which has been linked to impaired fetal brain development [26,27,28,29].

Regular physical activity has been demonstrated to enhance insulin sensitivity and facilitate the regulation of blood sugar levels. It is recommended that patients engage in activities such as walking, swimming, and prenatal yoga. Regular physical activity facilitates the effective utilization of insulin, which in turn reduces blood glucose levels. Engaging in at least 30 min of moderate exercise on a regular basis has been demonstrated to assist in regulating blood glucose levels and enhancing maternal health outcomes [30,31]. The evidence from small-scale studies indicates that physical activity can contribute to the reduction of blood glucose levels [31,32,33]. Adequate daily energy intake in conjunction with regular physical activity throughout the gestational period can ensure adequate weight gain during pregnancy based on maternal body mass index (BMI). This, in turn, can prevent adverse neonatal outcomes [26,27,31,32,33].

### 5.2. Pharmacological Control of Gestational Diabetes

Insulin is the most commonly prescribed pharmacological treatment for women with GDM who are unable to control their blood sugar levels through dietary and exercise modifications alone. Insulin is used to lower blood glucose levels by enhancing the body’s ability to use glucose and reducing the amount of glucose produced by the liver. The most usually administered insulins are rapid-acting (e.g., lispro, aspart) and long-acting (e.g., glargine, detemir), with the type and dosage adjusted to the patient’s blood glucose levels [27,29].

Metformin is an oral medication frequently employed as an adjunct to insulin or as an alternative in cases where insulin is not tolerated. Metformin exerts its therapeutic effects by enhancing insulin sensitivity and reducing glucose production in the liver. Metformin is classified as Category B by the FDA for use during pregnancy—the animal studies have not demonstrated any risk associated with its use during this period. However, the available evidence is limited to controlled human studies, which are insufficient to fully ascertain the safety of metformin during pregnancy. While there is no definitive evidence that metformin causes adverse effects on the fetus, the safety of metformin during pregnancy has not been sufficiently investigated to definitively exclude potential risks. Nevertheless, organizations, including the American Diabetes Association (ADA) and the International Diabetes Federation (IDF), have acknowledged the efficacy of metformin as a treatment option for women with GDM. The general findings of several randomized controlled trials (RCTs) and systematic reviews suggest that metformin is an effective means of controlling blood sugar levels and may reduce the need for insulin therapy in women with GDM, particularly in those who are overweight or obese. Studies comparing metformin with insulin for GDM management indicate that both medications are similarly effective in controlling glucose levels, although insulin is often considered the gold standard for GDM treatment. In certain instances, metformin has demonstrated comparable efficacy to insulin, while exhibiting fewer adverse effects, such as weight gain and hypoglycemia. Metformin crosses the placenta, but studies have not found any significant adverse effects on fetal development when used at doses typical of therapy for GDM. Research has shown that metformin does not cause major birth defects or fetal malformations. Some studies suggest that metformin does not increase the risk of fetal macrosomia (excessive fetal growth) or preterm birth compared with insulin therapy, although there are mixed results in the literature. There are also some concerns about the long-term effects of metformin exposure during pregnancy on offspring development, particularly with regard to neonatal metabolic outcomes (e.g., insulin resistance). However, these effects are not fully understood and long-term studies are ongoing [28,34,35,36].

Sulfonylureas (e.g., glyburide) are effective in controlling blood glucose levels in GDM and may be used when insulin therapy is not ideal. They have the potential for neonatal hypoglycemia and should be used with caution, particularly with regard to dosing and monitoring [28,34,35,36].

Acarbose is less commonly used in GDM but may be beneficial in controlling postprandial blood glucose levels. It is usually considered in milder cases or in combination with other medications. However, there is limited evidence on its safety and effectiveness in pregnancy. There are currently no published data on the use of other non-insulin drugs in pregnancy, such as dipeptidyl peptidyl 4 (DPP4) inhibitors, sodium-glucose co-transporter type 2 (SGLT2) inhibitors and glucagon-like peptide 1 (GLP1) receptor agonists [34,35,36].

## 6. Novel Biomarkers for the Pathogenesis of Diabetes

In addition to the well-established etiological factors associated with diabetes, the pathogenesis encompasses the impact of tissue hormones, including adipokines, on the regulation of glucose metabolism. This includes omentin-1 and visfatin, which are of particular interest to the authors, as well as leptin, resistin, adiponectin and TNF-α (Tumor Necrosis Factor-alpha). Adipokines are not only responsible for regulating metabolic processes and maintaining energy homeostasis; they also influence fetal development, primarily in regard to adipose tissue growth. The concentration and activity of these molecules depend on the visceral fat content of the mother’s body and the presence of coexisting metabolic disorders, including insulin resistance [9]. The effects of these molecules on insulin resistance, inflammation, and energy regulation can influence both maternal glucose metabolism and fetal growth. Dysregulated adipokine levels in GDM can result in adverse pregnancy outcomes, including fetal overgrowth, macrosomia, and long-term metabolic health issues in the offspring [37,38,39].

Omentin, also designated as intelectin, was initially identified in Paneth cells. Furthermore, in humans, it is produced in the lungs, intestines, and heart, and has also been demonstrated to be secreted by ovarian and placental cells. Omentin-1 is composed of 313 amino acids with a molecular weight of 33 kDa, yet its biological significance remains poorly understood [40]. It has been demonstrated that omentin concentrations are diminished in individuals with obesity, both in plasma and adipose tissue. Additionally, a positive correlation was observed between omentin-1 concentrations and those of adiponectin and high-density lipoprotein, while they were negatively correlated with body weight and indices of insulin resistance. Recombinant omentin has been demonstrated to enhance glucose uptake and insulin sensitivity in isolated adipocytes [41]. Furthermore, serum omentin-1 concentrations are diminished in children with type 1 diabetes in comparison to healthy children. Moreover, serum omentin-1 concentrations decline in correlation with the presence of the three major vascular complications of diabetes, including retinopathy, nephropathy, and neuropathy. The elevation of free omentin-1 concentrations is contingent upon weight loss, a diet replete with olive oil, aerobic exercise, and pharmacological intervention with antidiabetic agents, among other factors [40]. The data on serum omentin-1 concentrations in pregnant women with gestational diabetes mellitus (GDM) compared to healthy women remain inconclusive. While some studies have found no statistically significant differences between the two groups, others have reported lower serum omentin-1 concentrations in newborns from pregnancies complicated by GDM. This may increase the risk of developing insulin resistance in the future [42]. Alternatively, it has been demonstrated that maternal and cord serum concentrations are lower in pregnancies complicated by GDM. In this case, the hormone itself may serve as a predictor of preterm labor [43]. Furthermore, an increase in omentin-1 levels between 12 and 15 weeks of gestation was associated with a reduced risk of developing GDM, adjusted for maternal factors including BMI. Conversely, low omentin-1 levels in the first trimester of pregnancy were associated with a fourfold increased risk of developing gestational diabetes mellitus during pregnancy [44]. There is a paucity of data regarding the presence of omentin in breast milk and its correlation with body fat content.

Visfatin is also referred to as pre-B lymphocyte colony-stimulating factor (PBEF) or nicotinamide phosphoribosyltransferase (NAMPT). Visfatin is produced in a number of different cell types, including leukocytes, adipocytes, muscle cells (including those found in myometrial tissue), hepatocytes, placental cells, fetal membranes, and bone marrow cells [45]. The research on visfatin has yielded inconclusive results, and its role in the human body remains poorly understood. It is established that visfatin is an adipokine that plays a role in regulating energy homeostasis. For example, glucose concentration affects the secretion of visfatin by adipocytes [46], and the hormone itself binds to the insulin-1 receptor, exhibiting insulin-like effects, reduces glycogenolysis in hepatocytes, and stimulates glucose utilization in adipocytes and muscle cells [47]. Furthermore, it is implicated in the regulation of gene expression associated with oxidative stress and the inflammatory response [48,49]. Moreover, elevated serum visfatin levels have been documented in individuals with a body mass index (BMI) exceeding 30 kg/m^2^ and concomitant diagnoses of type 2 diabetes, metabolic syndrome, or cardiovascular disease [50], as well as in pregnant women with pregnancy-induced diabetes mellitus (PGDM) [51]. The concentration of visfatin has been observed to vary in women with gestational diabetes, with both higher and lower levels compared to healthy women. Furthermore, visfatin is known to be secreted into human milk. Its effects on fetal development, as well as on changes in infant weight and postpartum adipose tissue development, have been proposed [52,53].

In addition to its function in regulating appetite and maintaining energy balance, elevated leptin levels are frequently observed in GDM. It is postulated that elevated leptin concentrations may contribute to insulin resistance by interfering with insulin signaling pathways. Furthermore, elevated leptin levels during pregnancy have been linked to an increased risk of obesity. Leptin crosses the placenta and is essential for fetal growth and development. It plays a role in the regulation of fetal energy balance. However, excessive maternal leptin may result in fetal overgrowth and an increased risk of complications such as preterm birth or delivery complications. Moreover, elevated leptin levels may also exert an influence on the development of adipose tissue in the offspring [37,39].

Adiponectin is typically associated with insulin-sensitizing properties, which promote glucose uptake and fatty acid oxidation. Nevertheless, in women with GDM, adiponectin levels are frequently diminished, which contributes to insulin resistance. Adiponectin exerts an influence on fetal growth and placental function. Maternal levels of adiponectin that are below the optimal range are associated with an increased risk of fetal overgrowth and complications such as macrosomia. Adiponectin is thought to improve placental function and reduce inflammation, thereby the available data suggest that adiponectin plays an important role in fetal intrauterine development and growth during the early stages of life. However, the precise role of fetal adiponectin remains unclear [37,38,39].

Resistin is responsible for the promotion of insulin resistance. The available data suggest that resistin may exert some influence on insulin resistance during pregnancy, but it is likely to have a secondary role in the pathogenesis of GDM. However, it is postulated that elevated levels of resistin in gestational diabetes mellitus may serve to exacerbate the condition. Additionally, resistin can activate pathways involved in inflammatory processes, which further affects glucose regulation. The presence of elevated resistin levels in GDM may contribute to the occurrence of fetal growth disorders, which may result in augmented adipose tissue accumulation in the fetus and an elevated risk of developing metabolic diseases in later life [37,38,39].

Tumor necrosis factor-α (TNF-α), an inflammatory cytokine secreted by adipocytes, has been observed to be elevated in both obesity and GDM. It impairs insulin signaling and contributes to the development of insulin resistance. Elevated TNF-α in GDM can contribute to the development of an inflammatory response, which in turn affects placental function and fetal development. Chronic inflammation has the potential to impair placental nutrient transport and disrupt fetal growth patterns, which may ultimately result in adverse outcomes such as low birth weight or preterm birth [37,38,39].

Adipokines exert a direct and indirect influence on fetal growth and development, affecting maternal metabolism and placental function. The maternal and fetal adipokines and inflammatory biomarkers exerted a unique impact on the offspring’s anthropometrics during the initial postnatal year, irrespective of the maternal age, the pre-pregnancy body mass index (BMI) and the ethnicity [54]. A substantial proportion of the extant reviews of available studies posit that elevated maternal leptin and reduced adiponectin are associated with fetal overgrowth, driven by placental adaptation that modifies features including nutrient transporters and the function of placental mitochondria. Based on the available evidence, it is reasonable to conclude that these epigenetic adaptations in adipokines are likely to be functional in nature and may exert long-term influences on the regulation of energy metabolism in newborns. Maternal glycemia is associated with DNA methylation alterations at the gene loci of adipokines in the placenta, thereby implicating epigenetics in the programming of the newborn’s metabolic phenotype [37,38,39,55].

## 7. A Literature Review of the Impact of Gestational Diabetes on Fetal Growth and the Nutritional Status of the Newborn

Recent studies conducted in various regions of the world have confirmed that the occurrence of glucose metabolism disorders during pregnancy is associated with an increased risk of fetal macrosomia. Mothers with gestational diabetes, compared to healthy mothers, have been shown to give birth to heavier children significantly more often with a birth weight above the 90th percentile for fetal age (LGA newborns, or Large for Gestational Age) [56,57,58,59]. Immanuel et al. investigated the influence of the severity of glucose metabolism disorders on the risk of delivering an LGA newborn [56]. Women diagnosed with early gestational diabetes were classified into three groups based on their insulin resistance profiles: (1) GDM-R with insulin resistance above the median in the group (HOMA-IR ≥ 2.5 and Stumvoll index ≥ 1590), (2) GDM-S with insulin resistance below the median in the group (HOMA-IR < 2.5 and Stumvoll index < 1590), and (3) GDM-B with a combination of both above variants (HOMA-IR ≥ 2.5 and Stumvoll index < 1590). In comparison to women from the group with normal glucose tolerance, women from the GDM-R group exhibited elevated fasting and postprandial glucose levels, as well as elevated insulin concentrations. Furthermore, this group demonstrated an elevated risk of fetal macrosomia [OR 3.30, 95% CI 1.50–7.50] and a higher likelihood of undergoing cesarean delivery (adjusted OR 2.30, 95% CI 1.20–4.40). In the case of women from the GDM-S and GDM-B groups, the results of the analysis were comparable to those observed in the group of women with normal glucose tolerance [56].

The relationship between the type of gestational diabetes treatment and pregnancy complications was also assessed in the studies conducted on this topic to date. Fazel-Sarjoui et al. demonstrated that there were no statistically significant differences in the mean birth weight of newborns between the group of mothers with gestational diabetes who were treated with a diet and the group of mothers who were treated with insulin [60]. The two groups of women demonstrated comparable weight gain throughout the gestational period. Nevertheless, a lower incidence of premature births was observed in the cohort of mothers who received dietary treatment [60].

In a study conducted by Silva et al., all women diagnosed with gestational diabetes received nutritional consultation and advice on physical activity. They were then treated with a diet and lifestyle modifications, with regular glycemic monitoring and fetal anthropometric measurements assessed by ultrasound. In cases of mild gestational diabetes, metformin was introduced as a subsequent treatment option. If glycemic control remained inadequate, insulin therapy was initiated. In cases of severe gestational diabetes (defined as fasting glucose > 100 mg/dL, 1-h postprandial glucose > 140 mg/dL, or fetal abdominal circumference > the 90th percentile), insulin therapy was initiated promptly. The results of the study demonstrated that women who received metformin treatment were less likely to give birth to small-for-gestational-age (SGA) infants [OR 0.25, 95% CI 0.09–0.66] and more likely to give birth to eutrophic infants [OR 2.1, 95% CI 1.12–3.94]. The combination treatment with insulin and metformin was associated with an increased risk of LGA neonates [OR 3.57, 95% CI 1.14–11.15] and a decreased risk of preterm birth [OR 0.11, 95% CI 0.01–0.71]. The type of treatment did not affect the method of delivery, Apgar score, or the necessity for newborn intensive care unit (NICU) admission [61].

In a separate study by Koning et al. [57], the treatment of patients with gestational diabetes also commenced with dietary intervention under the guidance of a dietitian. In the event that fasting and postprandial glycemia remained uncontrolled after a period of 1–2 weeks, insulin was introduced as a supplementary treatment modality (long-acting insulin, prandial insulin, or a combination of both). The patients were required to perform control glucose measurements, maintain consistent communication with the medical staff, and undergo an ultrasound examination every four weeks to assess fetal growth. All women demonstrated effective glycemic control; however, the insulin-treated group exhibited elevated glycated hemoglobin levels. Furthermore, this cohort exhibited a higher prevalence of obesity, elevated glycemic values in the glucose tolerance test, an increased incidence of diabetes, and the delivery of a neonate with a body weight exceeding 4500 g in the preceding pregnancy. In comparison to the cohort treated with a dietary regimen, the group of women who received insulin therapy exhibited a markedly reduced mean birth weight of their infants, along with a diminished prevalence of macrosomic newborns (>4000 g). However, no notable discrepancies were observed between the two groups with respect to the percentile distribution of birth weight relative to fetal age. Furthermore, there were no significant differences in the incidence of LGA, SGA, or eutrophic newborns. The group of women treated with insulin exhibited a higher incidence of operative deliveries and cesarean section deliveries [57].

The birth weight of the newborn, including the risk of fetal macrosomia, may be contingent upon the mother’s weight gain during pregnancy [62,63]. Maayan-Metzger et al. demonstrated that infants born to mothers who exceeded the recommended weight gain norms exhibited higher birth weights and were more frequently delivered via cesarean section. Furthermore, these mothers were diagnosed with gestational diabetes mellitus, which required insulin therapy [62]. Wang et al. obtained similar study results, indicating that among the surveyed women with diagnosed gestational diabetes, excessive weight gain during pregnancy was a significant risk factor for fetal macrosomia (OR 2.884, 95% CI 1.385–6.004). Furthermore, high fasting glycemia [OR 1.933, 95% CI 1.126–3.316] and increased serum triglycerides in the third trimester of pregnancy [OR 1.235, 95% CI 1.053–1.449] were also found to significantly impact fetal development [63].

The severity of metabolic disorders during pregnancy and maternal weight gain determines the nutritional status of the newborn not only in terms of birth weight, but also body composition [64,65]. In a study conducted in Brazil, newborns of diabetic mothers had higher body fat content than newborns of healthy mothers, with higher body fat mass in boys than in girls. However, the main predictors of body fat content were maternal BMI before pregnancy [β 6.75; 95% CI 2.36–11.1] and weight gain during pregnancy [β 5.64; 95% CI 1.16–10.1] [64]. In contrast, a study conducted in Sweden found higher fat mass in girls born to women with gestational diabetes at both 1st and 12th weeks of age, compared to children of normal-weight healthy women. Overall, compared to data for the general Swedish population, these women gave birth to children of similar length but higher weight and BMI. In addition, a correlation was found between body fat percentage at 1st week of age and glycated hemoglobin value and fasting blood glucose in the third trimester of pregnancy [65].

## 8. The Long-Term Consequences for the Offspring of Mothers with GDM

As it has been already mentioned above, the offspring of mothers with GDM are at an elevated risk of developing a range of long-term health complications, particularly metabolic disorders such as obesity, type 2 diabetes, and cardiovascular disease. These risks are attributable, at least in part, to in utero exposure to altered glucose and insulin levels, which can lead to epigenetic changes and disruptions in normal development. The risks are influenced by several factors, including the severity of maternal hyperglycemia, the management of GDM, and genetic or environmental factors [26,66].

The incidence of childhood obesity is elevated among children born to mothers with GDM. This is a result of early exposure to elevated insulin levels prenatally, which is known to affect fat deposition and the regulation of appetite and metabolism. It has been also found that the risk of obesity is greater in children who are born with fetal macrosomia or whose mothers have poorly controlled blood glucose levels during pregnancy. These children typically exhibit elevated fat mass and altered body composition from an early age. However, the risk of obesity may be influenced by maternal lifestyle factors (e.g., diet, physical activity) during pregnancy [26,67,68].

One of the most significant long-term risks for children born to mothers with GDM is an increased likelihood of developing type 2 diabetes later in life, due to both genetic predisposition and early life exposure to hyperglycemia in utero. They are also at an elevated risk of developing insulin resistance and impaired glucose tolerance during childhood and adolescence, which potentially progress to the development of type 2 diabetes or metabolic syndrome (a cluster of conditions characterized by obesity, hypertension, hyperglycemia and dyslipidemia) in adulthood. These risks are further compounded by additional factors such as obesity, physical inactivity, and poor dietary habits, all of which can be influenced by both genetic and environmental factors [67,68]. The available evidence indicates that early exposure to maternal hyperglycemia and insulin resistance increases the likelihood of these conditions in the offspring. The mechanism is believed to be linked to in utero programming, whereby fetal development is affected by the metabolic environment of the mother, resulting in alterations to fetal gene expression, adipose tissue accumulation, and insulin sensitivity [26,66,67,68].

A number of studies have indicated that children born to mothers with GDM may be at an increased risk of developing cardiovascular diseases in later life, particularly if they also develop metabolic syndrome or obesity. The cardiovascular risk is likely attributable to a complex interplay between early life exposure to altered metabolic environments and genetic predisposition [69].

The evidence is mounting that GDM may have an impact on the cognitive and behavioral development of offspring, although the findings are not entirely consistent [70]. Some studies indicate that offspring may exhibit slightly lower IQ scores or an increased risk of learning disabilities or attention deficits [71]. This may be attributed to alterations in fetal brain development resulting from maternal hyperglycemia, omega-3 fatty acids and iron deficiency [70]. Nevertheless, other studies have not identified a clear association, and it is thought that factors such as maternal obesity, age and socioeconomic status may also influence the neurodevelopmental outcomes of these children [26,70,72,73].

All in all, with early monitoring, lifestyle interventions, and appropriate healthcare, many of these risks can be mitigated or managed to improve long-term health outcomes for offspring of mothers with GDM. In consideration of the aforementioned aspects pertaining to the effects of GDM on fetal development and the nutritional status of the newborn, it can be posited that hyperglycemia represents the most deleterious factor. This is due to its capacity to induce alterations in DNA methylation, impair protein translation, and consequently, give rise to aberrant production and impaired concentrations of cellular messengers. This is a pivotal factor influencing the nutritional and metabolic programming of the developing fetus, thereby predisposing the individual to the development of metabolic complications throughout their lifetime.

The aforementioned mechanisms appear to be corroborated by the findings of a recent Danish study [73]. The fetal growth rate was observed to be greater in women with GDM compared to those with normal glucose tolerance. The risk of overweight in children of mothers with GDM was higher too. Higher fetal growth rate in the third trimester could potentially explain some of the risk of overweight in the offspring of mothers with GDM. The accelerated fetal growth rate observed in the third trimester may have contributed to the increased risk of overweight in the offspring of mothers with GDM. Nevertheless, pre-pregnancy BMI also exerted a considerable influence on the risk of overweight in the offspring, indicating that additional factors, such as genetic predisposition, lifestyle, maternal hormonal factors and medications used to achieve glycemic control play a significant role in the risk of overweight in offspring affected by GDM [73,74].

## 9. Summary

The metabolic environment, resulting from abnormal glucose tolerance during pregnancy and its sequelae, exerts a particularly significant impact on fetal growth and, consequently, on the birth weight and fat mass of the newborn infants. The action of placental hormones (e.g., human placental lactogen, cortisol, and progesterone) on maternal glucose and lipid metabolism results in the exacerbation of hyperglycemia and the promotion of nutrient transfer and fetal overgrowth. The imbalanced availability of nutrients during gestation programs long-term metabolic pathways, which in turn impact energy homeostasis, insulin sensitivity, and appetite regulation. Thus, the process of intrauterine growth, including body composition after birth, in conjunction with genetic factors and intrauterine environment, serves to determine the risk of developing lifestyle diseases in the future. The early diagnosis and appropriate treatment of gestational diabetes mellitus can mitigate the adverse metabolic effects for both mother and newborn, disrupting intergenerational cycles of metabolic disorders. From a public health perspective, this can contribute to a reduction in healthcare costs and a decline in the prevalence of the condition and related chronic diseases within the population.

## References

[1] Wender-Ożegowska E., Bomba-Opoń D., Brązert J., Celewicz Z., Czajkowski K., Gutaj P., Malinowska-Polubiec A., Zawiejska A., Wielgoś M. (2018). Standards of Polish Society of Gynecologists and Obstetricians in management of women with diabetes [Standardy Polskiego Towarzystwa Ginekologów i Położników postępowania u kobiet z cukrzycą]. Ginekol. Pol..

[2] Baz B., Riveline J.P., Gautier J.F. (2016). Endocrinology of pregnancy: Gestational diabetes mellitus: Definition, aetiological and clinical aspects. Eur. J. Endocrinol..

[3] Manerkar K., Harding J., Conlon C., McKinlay C. (2020). Maternal gestational diabetes and infant feeding, nutrition and growth: A systematic review and meta-analysis. Br. J. Nutr..

[4] Mitanchez D., Yzydorczyk C., Siddeek B., Boubred F., Benahmed M., Simeoni U. (2015). The offspring of the diabetic mother—Short- and long-term implications. Best Pract. Res. Clin. Obstet. Gynaecol..

[5] Schwarzenberg S.J., Georgieff M.K., Committee on Nutrition (2018). Advocacy for Improving Nutrition in the First 1000 Days to Support Childhood Development and Adult Health. Pediatrics.

[6] Catalano P. (2002). The Diabetogenic State of Maternal Metabolism in Pregnancy. NeoReviews.

[7] Parrettini S., Caroli A., Torlone E. (2020). Nutrition and Metabolic Adaptations in Physiological and Complicated Pregnancy: Focus on Obesity and Gestational Diabetes. Front. Endocrinol..

[8] Plows J.F., Stanley J.L., Baker P.N., Reynolds C.M., Vickers M.H. (2018). The Pathophysiology of Gestational Diabetes Mellitus. Int. J. Mol. Sci..

[9] Herrera E., Ortega-Senovilla H. (2010). Maternal lipid metabolism during normal pregnancy and its implications to fetal development. Clin. Lipidol..

[10] Herrera E., Desoye G. (2016). Maternal and fetal lipid metabolism under normal and gestational diabetic conditions. Horm. Mol. Biol. Clin. Investig..

[11] Barrett H.L., Dekker Nitert M., McIntyre H.D., Callaway L.K. (2014). Normalizing metabolism in diabetic pregnancy: Is it time to target lipids?. Diabetes Care.

[12] Herrera E., Djelmiš J., Desoye G., Ivaniševic M. (2005). Metabolic changes in diabetic pregnancy. Diabetology of Pregnancy.

[13] Butte N.F. (2000). Carbohydrate and lipid metabolism in pregnancy: Normal compared with gestational diabetes mellitus. Am. J. Clin. Nutr..

[14] Pawlik D., Radziszewska R. (2015). The maternal diabetes mellitus and consequences for newborn. Endokrynol. Ped..

[15] Świątoniowska N., Rozensztrauch A. (2017). The influence of gestational diabetes mellitus on the developing baby. J. Educ. Health Sport.

[16] Hiden U., Glitzner E., Hartmann M., Desoye G. (2009). Insulin and the IGF system in the human placenta of normal and diabetic pregnancies. J. Anat..

[17] Sferruzzi-Perri A.N., Owens J.A., Pringle K.G., Roberts C.T. (2011). The neglected role of insulin-like growth factors in the maternal circulation regulating fetal growth. J. Physiol..

[18] Kaur H., Muhlhausler B.S., Roberts C.T., Gatford K.L. (2021). The growth hormone-insulin like growth factor axis in pregnancy. J. Endocrinol..

[19] Umana-Perez A., Novoa-Herran S., Castro J., Correa-Sanchez A., Guevara V., Lopez-Gonzalez D., Sanchez-Gomez M. (2020). Role of the Insulin-like growth factor axis and the Transforming growth factor-β in the regulation of the placenta and the pathogenesis of Gestational Trophoblastic Diseases. Med. Res. Arch..

[20] Thornton J.M., Shah N.M., Lillycrop K.A., Cui W., Johnson M.R., Singh N. (2024). Multigenerational diabetes mellitus. Front. Endocrinol..

[21] Calvo M.J., Parra H., Santeliz R., Bautista J., Luzardo E., Villasmil N., Martínez M.S., Chacín M., Cano C., Checa-Ros A. (2024). The Placental Role in Gestational Diabetes Mellitus: A Molecular Perspective. Eur. Endocrinol..

[22] Castillo-Castrejon M., Powell T.L. (2017). Placental Nutrient Transport in Gestational Diabetic Pregnancies. Front. Endocrinol..

[23] Stern C., Schwarz S., Moser G., Cvitic S., Jantscher-Krenn E., Gauster M., Hiden U. (2021). Placental Endocrine Activity: Adaptation and Disruption of Maternal Glucose Metabolism in Pregnancy and the Influence of Fetal Sex. Int. J. Mol. Sci..

[24] Olmos-Ortiz A., Flores-Espinosa P., Díaz L., Velázquez P., Ramírez-Isarraraz C., Zaga-Clavellina V. (2021). Immunoendocrine Dysregulation during Gestational Diabetes Mellitus: The Central Role of the Placenta. Int. J. Mol. Sci..

[25] Carrasco-Wong I., Moller A., Giachini F.R., Lima V.V., Toledo F., Stojanova J., Sobrevia L., San Martín S. (2020). Placental structure in gestational diabetes mellitus. Biochim. Biophys. Acta Mol. Basis Dis..

[26] Huang J., Wu Y., Li H., Cui H., Zhang Q., Long T., Zhang Y., Li M. (2023). Weight Management during Pregnancy and the Postpartum Period in Women with Gestational Diabetes Mellitus: A Systematic Review and Summary of Current Evidence and Recommendations. Nutrients..

[27] Wei X., Zou H., Zhang T., Huo Y., Yang J., Wang Z., Li Y., Zhao J. (2024). Gestational Diabetes Mellitus: What Can Medical Nutrition Therapy Do?. Nutrients.

[28] Mukherjee S.M., Dawson A. (2022). Diabetes: How to manage gestational diabetes mellitus. Drugs Context.

[29] Mitanchez D., Ciangura C., Jacqueminet S. (2020). How Can Maternal Lifestyle Interventions Modify the Effects of Gestational Diabetes in the Neonate and the Offspring? A Systematic Review of Meta-Analyses. Nutrients.

[30] Gestational Diabetes Mellitus (2018). ACOG Practice Bulletin No. 190. Obstet. Gynecol..

[31] Mottola M.F., Artal R. (2016). Fetal and maternal metabolic responses to exercise during pregnancy. Early Hum. Dev..

[32] Anjana R.M., Sudha V., Lakshmipriya N., Anitha C., Unnikrishnan R., Bhavadharini B., Mahalakshmi M.M., Maheswari K., Kayal A., Ram U. (2016). Physical activity patterns and gestational diabetes outcomes—The wings project. Diabetes Res. Clin. Pract..

[33] Russo L.M., Nobles C., Ertel K., Chasan-Taber L., Whitcomb B.W. (2015). Physical activity interventions in pregnancy and risk of gestational diabetes mellitus. Obstet. Gynecol..

[34] Radenković M., Jakovljević A. (2022). Pharmacotherapy of Gestational Diabetes Mellitus: Current Recommendations. Gestational Diabetes Mellitus—New Developments.

[35] Skóra A., Hajduk-Maślak K., Galasińska I., Michalik B., Szypuła A., Sęk M. (2024). Gestational diabetes—Management strategies including pharmacological treatment and lifestyle interventions. J. Educ. Health Sport.

[36] Mukerji G., Feig D.S. (2017). Pharmacological Management of Gestational Diabetes Mellitus. Drugs.

[37] Karami M., Mousavi S.H., Rafiee M., Heidari R., Shahrokhi S.Z. (2023). Biochemical and molecular biomarkers: Unraveling their role in gestational diabetes mellitus. Diabetol. Metab. Syndr..

[38] Mallardo M., Ferraro S., Daniele A., Nigro E. (2021). GDM-complicated pregnancies: Focus on adipokines. Mol. Biol. Rep..

[39] Kabbani N., Blüher M., Stepan H., Stumvoll M., Ebert T., Tönjes A., Schrey-Petersen S. (2023). Adipokines in Pregnancy: A Systematic Review of Clinical Data. Biomedicines.

[40] Watanabe T., Watanabe-Kominato K., Takahashi Y., Kojima M., Watanabe R. (2017). Adipose Tissue-Derived Omentin-1 Function and Regulation. Compr. Physiol..

[41] Jaganathan R., Ravindran R., Dhanasekaran S. (2018). Emerging Role of Adipocytokines in Type 2 Diabetes as Mediators of Insulin Resistance and Cardiovascular Disease. Can. J. Diabetes.

[42] Franz M., Polterauer M., Springer S., Kuessel L., Haslinger P., Worda C., Worda K. (2018). Maternal and neonatal omentin-1 levels in gestational diabetes. Arch. Gynecol. Obstet..

[43] Mierzyński R., Dłuski D., Nowakowski Ł., Poniedziałek-Czajkowska E., Leszczyńska-Gorzelak B. (2018). Adiponectin and Omentin Levels as Predictive Biomarkers of Preterm Birth in Patients with Gestational Diabetes Mellitus. Biomed. Res. Int..

[44] Abell S.K., Shorakae S., Harrison C.L., Hiam D., Moreno-Asso A., Stepto N.K., De Courten B., Teede H.J. (2017). The association between dysregulated adipocytokines in early pregnancy and development of gestational diabetes. Diabetes Metab. Res. Rev..

[45] Deng Y., Scherer P.E. (2010). Adipokines as novel biomarkers and regulators of the metabolic syndrome. Ann. N. Y. Acad. Sci..

[46] Liang Z., Wu Y., Xu J., Fang Q., Chen D. (2016). Correlations of serum visfatin and metabolisms of glucose and lipid in women with gestational diabetes mellitus. J. Diabetes Investig..

[47] Adeghate E. (2008). Visfatin: Structure, function and relation to diabetes mellitus and other dysfunctions. Curr. Med. Chem..

[48] El-Taweel H.M.A., Salah N.A., Selem A.K., El-Refaeey A.A., Abdel-Aziz A.F. (2018). Visfatin gene expression and oxidative stress in pregnancy induced hypertension. Egypt. J. Basic Appl. Sci..

[49] Marseglia L., D’Angelo G., Manti M., Arrigo T., Barberi I., Reiter R.J., Gitto E. (2016). Visfatin: New marker of oxidative stress in preterm newborns. Int. J. Immunopathol. Pharmacol..

[50] Chang Y.H., Chang D.M., Lin K.C., Shin S.J., Lee Y.J. (2011). Visfatin in overweight/obesity, type 2 diabetes mellitus, insulin resistance, metabolic syndrome and cardiovascular diseases: A meta-analysis and systemic review. Diabetes Metab. Res. Rev..

[51] Coskun A., Ozkaya M., Kiran G., Kilinc M., Arikan D.C. (2010). Plasma visfatin levels in pregnant women with normal glucose tolerance, gestational diabetes and pre-gestational diabetes mellitus. J. Matern. Fetal Neonatal Med..

[52] Mashhad Taraqi A.S., Tehranian N., Roudbaneh S.P., Esmaeilzadeh M.S., Kazemnejad A., Aghoozi M.F., Yousefi S. (2018). Visfatin as a predictor for growth of fetus and infant. Turk. J. Obstet. Gynecol..

[53] Bienertová-Vašků J., Bienert P., Zlámal F., Tomandl J., Tomandlová M., Dostálová Z., Vašků A. (2012). Visfatin is secreted into the breast milk and is correlated with weight changes of the infant after the birth. Diabetes Res. Clin. Pract..

[54] Antoniou M.C., Quansah D.Y., Gilbert L., Arhab A., Schenk S., Lacroix A., Stuijfzand B., Horsch A., Puder J.J. (2024). Association between maternal and fetal inflammatory biomarkers and offspring weight and BMI during the first year of life in pregnancies with GDM: MySweetheart study. Front. Endocrinol..

[55] Downs T., da Silva Costa F., de Freitas Paganoti C., Holland O.J., Hryciw D.H. (2024). Adiponectin and Leptin during Pregnancy: A Systematic Review of Their Association with Pregnancy Disorders, Fetal Growth and Placental Function. Endocrines.

[56] Immanuel J., Simmons D., Harreiter J. (2021). Metabolic phenotypes of early gestational diabetes mellitus and their association with adverse pregnancy outcomes. Diabet. Med..

[57] Koning S.H., Hoogenberg K., Scheuneman K.A., Baas M.G., Korteweg F.J., Sollie K.M., Schering B.J., van Loon A.J., Wolffenbuttel B.H.R., van den Berg P. (2016). Neonatal and obstetric outcomes in diet- and insulin-treated women with gestational diabetes mellitus: A retrospective study. BMC Endocr. Disord..

[58] Riskin A., Itzchaki O., Bader D., Iofe A., Toropine A., Riskin-Mashiah S. (2020). Perinatal Outcomes in Infants of Mothers with Diabetes in Pregnancy. Isr. Med. Assoc. J..

[59] Verd S., de Sotto D., Fernández C., Gutiérrez A. (2016). The Effects of Mild Gestational Hyperglycemia on Exclusive Breastfeeding Cessation. Nutrients.

[60] Fazel-Sarjoui Z., Khodayari Namin A., Kamali M., Khodayari Namin N., Tajik A. (2016). Complications in neonates of mothers with gestational diabetes mellitus receiving insulin therapy versus dietary regimen. Int. J. Reprod. Biomed..

[61] Silva A.L., Amaral A.R., Oliveira D.S., Martins L., Silva M.R., Silva J.C. (2017). Neonatal outcomes according to different therapies for gestational diabetes mellitus. J. Pediatr..

[62] Maayan-Metzger A., Schushan-Eisen I., Strauss T., Globus O., Leibovitch L. (2015). Gestational weight gain and body mass indexes have an impact on the outcomes of diabetic mothers and infants. Acta Paediatr..

[63] Wang N., Ding Y., Wu J. (2018). Effects of pre-pregnancy body mass index and gestational weight gain on neonatal birth weight in women with gestational diabetes mellitus. Early Hum. Dev..

[64] Abreu L.R.S., Shirley M.K., Castro N.P., Euclydes V.V., Bergamaschi D.P., Luzia L.A., Cruz A.M., Rondó P.H.C. (2019). Gestational diabetes mellitus, pre-pregnancy body mass index, and gestational weight gain as risk factors for increased fat mass in Brazilian newborns. PLoS ONE.

[65] Andersson-Hall U.K., Järvinen E.A.J., Bosaeus M.H., Gustavsson C.E., Hårsmar E.J., Niklasson C.A., Albertsson-Wikland K.G., Holmäng A.B. (2019). Maternal obesity and gestational diabetes mellitus affect body composition through infancy: The PONCH study. Pediatr. Res..

[66] Moon J.H., Jang H.C. (2022). Gestational Diabetes Mellitus: Diagnostic Approaches and Maternal-Offspring Complications. Diabetes Metab. J..

[67] Meek C.L. (2023). An unwelcome inheritance: Childhood obesity after diabetes in pregnancy. Diabetologia.

[68] Kaul P., Bowker S.L., Savu A., Yeung R.O., Donovan L.E., Ryan E.A. (2019). Association between maternal diabetes, being large for gestational age and breast-feeding on being overweight or obese in childhood. Diabetologia.

[69] Chen A., Tan B., Du R., Chong Y.S., Zhang C., Koh A.S., Li L.J. (2024). Gestational diabetes mellitus and development of intergenerational overall and subtypes of cardiovascular diseases: A systematic review and meta-analysis. Cardiovasc. Diabetol..

[70] Rodolaki K., Pergialiotis V., Iakovidou N., Boutsikou T., Iliodromiti Z., Kanaka-Gantenbein C. (2023). The impact of maternal diabetes on the future health and neurodevelopment of the offspring: A review of the evidence. Front. Endocrinol..

[71] Chen K.R., Yu T., Lien Y.J., Chou Y.Y., Kuo P.L. (2023). Childhood neurodevelopmental disorders and maternal diabetes: A population-based cohort study. Dev. Med. Child. Neurol..

[72] Titmuss A., D’Aprano A., Barzi F., Brown A.D.H., Wood A., Connors C., Boyle J.A., Moore E., O’Dea K., Oats J. (2022). Hyperglycemia in pregnancy and developmental outcomes in children at 18–60 months of age: The PANDORA Wave 1 study. J. Dev. Orig. Health Dis..

[73] Leth-Møller M., Hulman A., Kampmann U., Hede S., Ovesen P.G., Knorr S. (2024). Effect of gestational diabetes on fetal growth rate and later overweight in the offspring. J. Clin. Endocrinol. Metab..

[74] Mirabelli M., Chiefari E., Tocci V., Greco E., Foti D., Brunetti A. (2021). Gestational diabetes: Implications for fetal growth, intervention timing, and treatment options. Curr. Opin. Pharmacol..

